# Endomembrane remodeling and dynamics in *Salmonella* infection

**DOI:** 10.15698/mic2022.02.769

**Published:** 2021-12-27

**Authors:** Ziyan Fang, Stéphane Méresse

**Affiliations:** 1 Aix Marseille University, CNRS, INSERM, CIML, Marseille, France.

**Keywords:** Salmonella, molecular motors, type 3 secretion system, effectors

## Abstract

*Salmonella*e are bacteria that cause moderate to severe infections in humans, depending on the strain and the immune status of the infected host. These pathogens have the particularity of residing in the cells of the infected host. They are usually found in a vacuolar compartment that the bacteria shape with the help of effector proteins. Following invasion of a eukaryotic cell, the bacterial vacuole undergoes maturation characterized by changes in localization, composition and morphology. In particular, membrane tubules stretching over the microtubule cytoskeleton are formed from the bacterial vacuole. Although these tubules do not occur in all infected cells, they are functionally important and promote intracellular replication. This review focuses on the role and significance of membrane compartment remodeling observed in infected cells and the bacterial and host cell pathways involved.

## INTRODUCTION

There are two species of *Salmonella* (*S*. enterica and *S*. bongori) but the strains that are pathogenic to humans almost always belong to the species enterica, which is subdivided into six subspecies and a large number of serotypes. *Salmonella* infections in humans are classified as typhoid or non-typhoid [1]. The former are serious systemic infections caused by *S*. enterica Typhi or Paratyphi and must be treated with antibiotics. The latter are usually epidemic gastroenteritis caused most of the times by *S*. Enteritidis or *S*. Typhimurium. Millions of people are infected with *Salmonella* each year and the annual cost of these infections is in the billions of dollars [2, 3].

Salmonellosis is a food-borne disease. After ingestion, these bacteria have little resistance to the extremely acidic environment of the stomach [4]. Those that survive reach the intestinal tract. Although confronted with the intestinal homeostatic barrier consisting of numerous antimicrobial molecules, *Salmonella* infects the gut epithelial cells and causes acute local inflammation. The latter produces reactive oxygen species that react with endogenous sulphur compounds forming a new electron acceptor, tetrathionate, which *Salmonella* is able to use for respiration [5]. This characteristic gives *Salmonella* a growth advantage over the host microbiota. Whether the infection is localized or systemic depends on the serovar-host association, but little is known about the molecular basis that dictates the outcome of the infection [6, 7]. In the case of gastroenteritis (e.g. *S*. Typhimurium in humans), there is a massive influx of neutrophils that migrate through the epithelium in response to the secretion of proinflammatory cytokines by the epithelial cells. These neutrophils phagocytose *Salmonella*e and the inflammatory diarrhea they cause clears the infection. In a systemic infection model (*S*. Typhimurium in mice), there is also an influx of neutrophils, but this is insufficient to control the infection. It has been proposed that this is related to the ability of *Salmonella* to rapidly infect epithelial cells and then immune system cells and thus protect itself from the inflammatory response [8].

*Salmonella* can cross the enterocyte barrier in different ways. Microfold (M) cells in Peyers’ patches, although few in number, are a preferential entry site [9]. Their number increases during infection as a result of the activity of an effector protein, secreted by *Salmonella*, which triggers the differentiation of epithelial cells into M cells and promotes host colonization [10]. Dendritic cells are also major players in *Salmonella* infection. Those present in the subepithelial region use their ability to emit dendrites that pass between enterocytes without breaking the integrity of the epithelial barrier [11] or through M cells [12] and capture the bacteria present in the intestinal lumen. Bacteria that have crossed the enteric barrier can migrate to the mesenteric lymph nodes, cross the gut-vascular barrier [13] and spread through the bloodstream, causing a systemic infection. It should be noted that the quality of the host’s adaptive response is also critical to the outcome of the infection, which is very problematic in countries with a high prevalence of immunosuppressive infections (HIV and/or parasitic infections). In such cases, frequent systemic infections with non-typhoidal *Salmonella* strains are observed [14].

## *SALMONELLA* IS AN INTRACELLULAR PATHOGEN

An intracellular pathogen is an organism whose virulence depends on the ability to replicate in a host cell. The demonstration of a causal link between the inability to multiply intracellularly and the profound attenuation of certain mutant strains has characterized *Salmonella* as such. Although documented as early as 1967 [15], the significance, importance and mechanisms underlying the presence of *Salmonella* in intestinal epithelial cells were not understood until much later. The development of *ex vivo* infection systems using cultured HeLa cells [16] and the discovery of *Salmonella* pathogenicity islands 1 (SPI-1 [17]) and 2 (SPI-2 [18, 19]) have been essential in this respect. SPI-1 and -2 are large gene clusters that code for distinct type III secretion systems, named T3SS-1 and T3SS-2. These are needle-like apparatus that cross the inner and outer membranes of the bacterium, project outwards and terminate in an oligomeric protein structure that forms a pore in the host cell membrane (plasma membrane or intracellular vacuole) and which *Salmonella* uses to transfer virulence factors from its cytoplasm to that of the host cell [20, 21]. Although structurally very similar, T3SS-1 and 2 are expressed at distinct phases of infection and have very different substrate repertoires. Their functions are essential at different stages of infection and largely independent of each other. SPI-1 is in several respects important during the intestinal phase of the infection. Its activity triggers an inflammatory response in the intestinal mucosa that promotes the growth of *Salmonella* over commensal bacteria and overcomes the colonization resistance that the microbiota induces ([22] and for review [23]). Another key function of SPI-1 is to allow *Salmonella* to invade non-phagocytic cells, in this specific case enterocytes, and thus pass the intestinal barrier. This is best illustrated by the fact that, in the mouse model of infection, an orally administered SPI-1 mutant is attenuated but fully virulent if injected intraperitoneally [24]. In the same model, the virulence of a SPI-2 mutant is greatly reduced whether it is orally or intraperitoneally inoculated, showing that SPI-2 is a major virulence factor for the systemic phase of infection [18, 19]. However, SPI-2 is also involved in the intestinal phase of the infection as it has been shown to be necessary for the bacteria to reach the basolateral side of the enterocytes and thus cross the intestinal barrier [25]. SPI-2 expression is induced inside host cells [26] and is essential for intracellular replication [27, 28]. Thus, the ability to replicate intracellularly is essential to the virulence of this bacterium.

During the infectious process, *Salmonella* will encounter and infect a large number of different cell types. The first will be the epithelial enterocytic cells and then rapidly, as soon as the intestinal barrier is crossed, the cells of the immune system (dendritic cells [29], neutrophils [30], B cells [31], macrophages [32]) but also fibroblasts [33] or epithelial cells of the gall bladder [34]. Within the cell, *Salmonella* adopts a vacuolar mode of life but in some cells or circumstances the bacteria may be present in the cytosol.

## MATURATION OF THE *SALMONELLA*-CONTAINING VACUOLE

### Formation of the *Salmonella* vacuole

**Figure 1:**
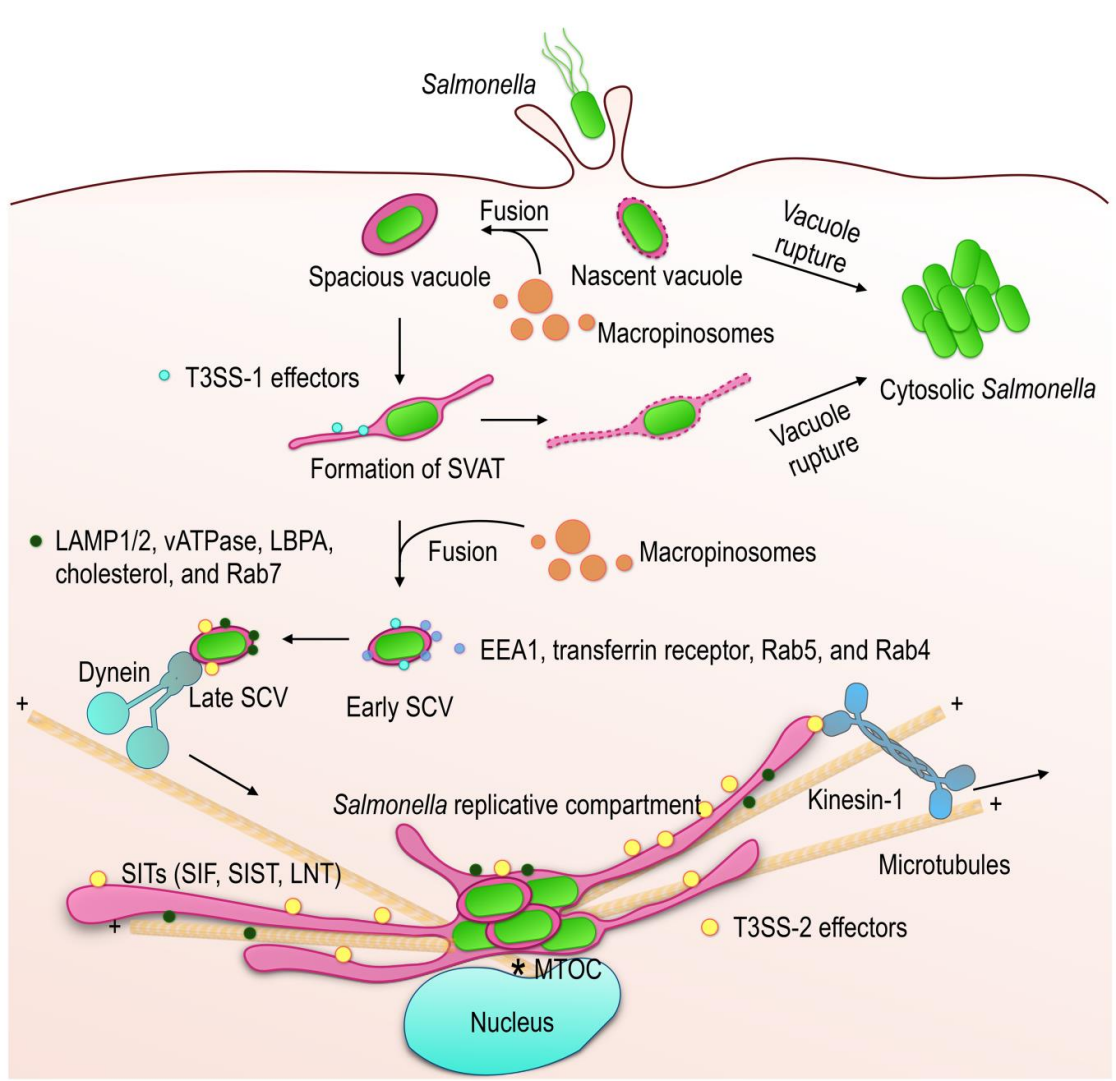
FIGURE 1: Maturation of the *Salmonella*-containing vacuole. *Salmonella* is able to infect non-phagocytic cells through the activity of effectors injected into the cell by T3SS-1. The formation of membrane ruffles allows entry into the cell and induces the formation of macropinosomes. The nascent vacuole is weakened by the presence of T3SS-1 and prone to rupture (5–20% of bacteria). *Salmonella* replicates rapidly in the cytosol of epithelial cells. Fusion of the nascent vacuole with macropinosomes increases its volume (spacious vacuole) and stabilizes it. Spacious vacuoles undergo further membrane remodeling, including the formation of SVAT with the help of the T3SS-1 effectors (south sea blue dots) and the host proteins SNX1/3. SVAT formation promotes vacuole rupture if membrane loss is not compensated by fusion activity with macropinosomes. The Early SCV is characterized by the presence of early endosomal markers (light blue dots). Its maturation is marked by the loss of early endosomal markers, the acquisition of late endosomal markers (dark green dots) and a shift of the SCV from the cell periphery to the MTOC region. Changes in the content and physico-chemical properties of the SCV, in particular the drop in its pH, induce the expression of T3SS-2 and its effectors (yellow dots). These allow the establishment of the replicative compartment whose membrane is very similar to that of the lysosomes. During the replication phase, the SCVs remain in the juxtanuclear region and are associated with membrane tubules (SITs), of the same composition, that stretch into the cell, supported by the microtubules and under the action of associated molecular motors.

To enter a host cell, *Salmonella* can undergo phagocytosis when the host cell is capable of doing so, or use an active mechanism and induce its internalization. In the latter case, once in contact with the host cell surface, *Salmonella* injects effector proteins using the T3SS-1. The T3SS-1 effectors promote the local polymerization of actin filaments, the formation of membrane ruffles which, by sealing around the bacteria, cause their internalization (**Fig. 1**) [35]. Effectors stimulate this phenomenon by acting on the Rho GTPases that regulate actin polymerization or by decreasing the critical concentration of actin required for polymerization (for review see [23]). *Salmonella* can invade macrophages in a T3SS-1-dependent manner or by being phagocytosed. However, the fate of the infected cell and that of the bacteria are very different, on the one hand because T3SS-1 activity initiates in macrophages a programmed cell death by apoptosis [36] and on the other hand, because the mode of entry impacts the ability of *Salmonella* to replicate intracellularly [37]. After passive or induced internalization, *Salmonella* is found in a vacuolar compartment which is commonly called *Salmonella*-containing vacuole (SCV).

In 1994, Alpuche-Aranda *et al*. noted that *Salmonellae* in contact with bone marrow-derived macrophages are found in large SCVs, which they called spacious phagosomes [38], and observed the contiguous presence of macropinosomes (**Fig. 1**). In the same year, a similar phenomenon was described during the invasion of epithelial cells [39]. The nascent SCVs, macropinosomes and membrane ruffles caused by *Salmonella* are rich in phosphatidylinositol-3-phosphate (PtdIns(3)P). The presence of this phosphoinositide is dependent on *Salmonella* since it is still present in cells treated with PtdIns 3-kinase inhibitors whereas it disappears from the early endosomes on which it is specifically present [40]. It was later shown to be dependent on the T3SS-1 effector SopB whose inositol phosphate phosphatase activity is necessary for the formation of macropinosomes and the maintenance of high levels PtdIns(3)P in the membrane of SCVs [41]. More recently, it was found that the bacterium is initially located in a tight SCV and that the spacious vacuole forms by the fusion of the nascent SCV with surrounding macropinosomes [42], a process controlled by the SNARE proteins SNAP25 and STX4 (**Fig. 1**) [43]. The initial vacuoles undergo further membrane remodeling, including the formation of spacious vacuole-associated tubules (SVAT; **Fig. 1**). These tubules are characterized by the presence of sorting nexin (SNX) 1 [44] and 3 [45], which are necessary for their formation. SVAT formation leads to vacuolar shrinkage and promotes vacuole rupture if not compensated by macropinosome fusion (**Fig. 1**) [43]. Conversely, this promotes the exclusion of the mannose-6 phosphate receptors (M6PR, the function of M6PRs is described in a subsequent paragraph) from SCVs [44] and probably contributes to limiting the presence of lysosomal enzymes.

### The nascent vacuole of *Salmonella* is susceptible to rupture

*Salmonella* has long been regarded as an intracellular bacterium with a vacuolar lifestyle. Data from the last ten years have changed this perception by showing that in some cells, the bacteria replicate in the cytosol and that this lifestyle may play a role in the pathophysiology of the infection.

The nascent vacuole is unstable but its level of instability depends on a large number of factors. It has been estimated that in epithelial cells and depending on the cell line used, between 5 and 20% of bacteria are released from the vacuole into the host cytosol during the first hours of infection [46]. This number may also vary depending on the multiplicity of the infection and the *Salmonella* strain [47]. In the early stages of infection, and as seen above, vacuole stability depends on the balance of membrane fluxes affected by the formation of SVATs and the fusion of SCVs and macropinosomes (**Fig. 1**). The nascent vacuole is also destabilized by the presence of T3SS-1. It allows the delivery of effectors into the host cell but also damages and weakens the host membrane [46]. The same goes for some of its effectors, SopE for example, an enzyme whose activity weakens the membrane of the nascent vacuole [47]. However, membrane repair mechanisms promoting intravacuolar retention of *Salmonella* but with different outcomes have been described. The first one described occurs in macrophages in response to an influx of calcium from the damaged SCV into the cytosol and triggers a synaptotagmin VII-dependent fusion of the injured bacterial vacuole with lysosomes. This process limits intracellular bacterial replication, presumably by inducing a massive arrival of degradative enzymes [48]. Another mechanism involves autophagy in the repair of host membranes damaged by T3SS-1 and promotes the retention and intravacuolar replication of *Salmonella* [49]. This is quite distinct from antibacterial autophagy, which follows the binding of Galectin 8 to damaged SCVs [50].

**Figure 2:**
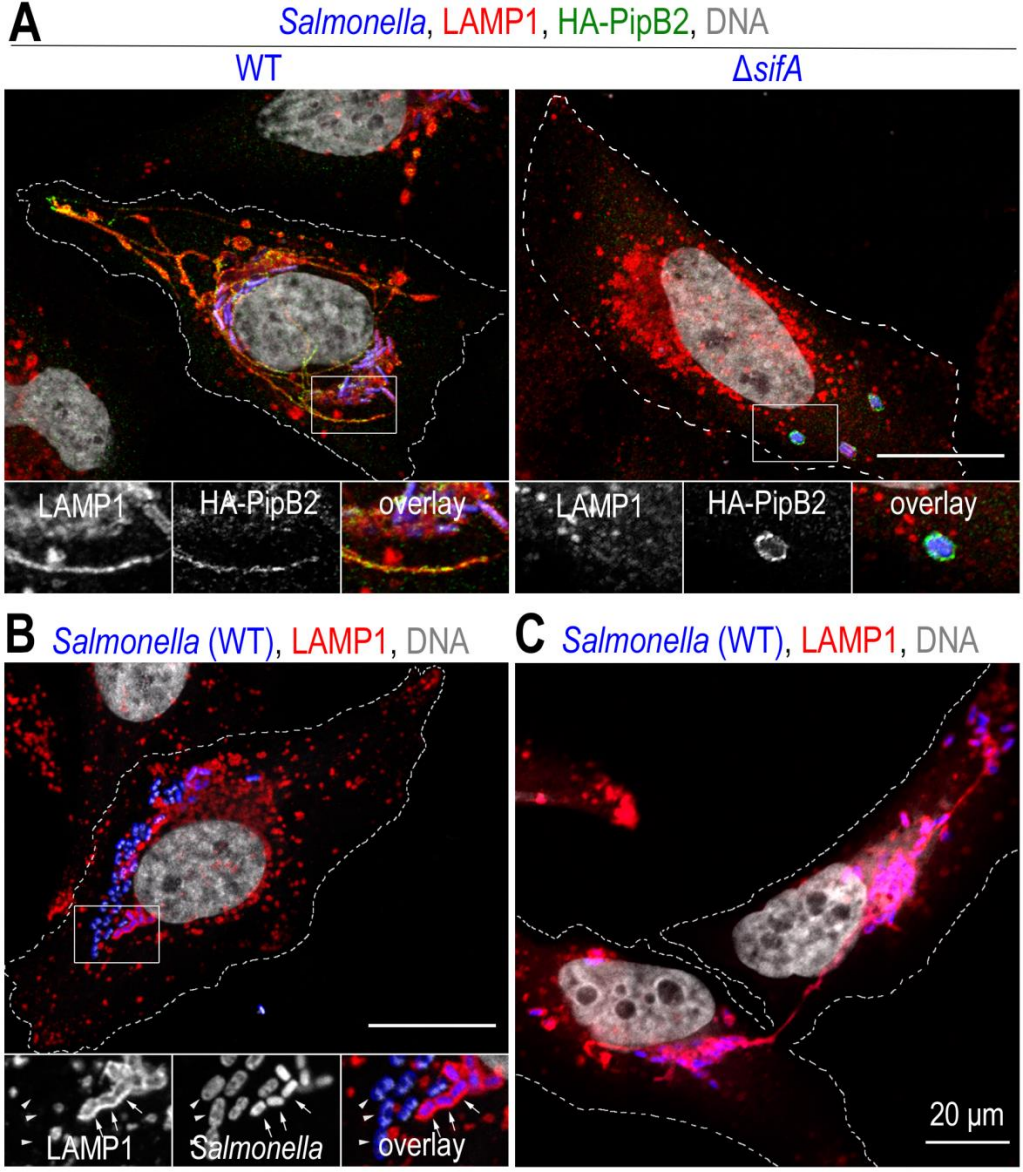
FIGURE 2: *Salmonella* induces a reorganization of the lysosomal compartment of the host cell. Confocal microscopy images, after immunolabelling, illustrating phenotypes observed in HeLa cells at a late time (12 h) of infection. (**A**) In cells infected with wild-type *Salmonella* (WT), the presence of bacteria (blue) in the juxtanuclear region is observed. The bacteria are in a vacuolar compartment (SCV) with a membrane similar to that of lysosomes, illustrated here by LAMP1 labelling (red). Membrane tubules (SITs) stretching to the cell periphery are very characteristic of *Salmonella*-infected HeLa cells. Some effectors secreted by T3SS-2, here HA-tagged PipB2 (green), are present on SCVs and SITs. In the absence of SifA (Δ*sifA*), the lysosome distribution of the infected cell appears to be little or not at all impaired. SCVs, which very often enclose several Δ*sifA* bacteria, adopt a position midway between the nucleus and the plasma membrane and are characterized by very weak LAMP1 labelling but strong labelling for membrane effectors (PipB2). (**B**) *Salmonella* is commonly found in the cytosol of epithelial cells. The image shows bacteria that are not surrounded by LAMP1 labelling (arrowheads) and are larger than those present in SCVs (arrows). (**C**) The image illustrates the distribution of SCVs in the two sister cells following mitosis and the fact that a SIT connects the two groups of bacteria until the two cells separate. Scale bar, 20 or 10 μm for the magnified insets. This figure is composed of unpublished images.

Once in the cytosol of epithelial cells (**Fig. 2B**), *Salmonella* multiplies rapidly [51], a phenomenon called hyper-replication [34] that is not observed in macrophages. *Salmonellae* that pass into the cytosol are, in macrophages, confronted with deleterious activities and therefore rapidly eliminated. Cytosolic bacteria are polyubiquitinated and targeted by the proteasome in macrophages but not in epithelial cells [52]. Bactericidal activity has been identified in the cytosol of macrophages [51] but it is not known whether it is related to bacterial recruitment of the proteasome, to the proteolytic activities of caspase-1 and caspase-11 [53] or to a combination of these and other as yet unknown activities.

Although it concerns only a limited fraction of the bacteria and is restricted to epithelial cells, cytosolic replication of *Salmonella* plays a role in infection. The presence of cytosolic *Salmonella* has been shown in gallbladder epithelial cells of infected mice [34] and in bovine and mouse enterocytes [54, 55]. The cellular response to this presence is the extrusion of infected cells from the monolayer. This phenomenon exists outside the context of infection but is strongly exacerbated in the presence of *Salmonella*. It releases bacteria expressing T3SS-1 into the lumen of the gallbladder or directly into the intestinal lumen, and these are then eliminated if they remain contained in the cell volume or, as has been shown in the gallbladder, may be free and participate in maintaining the infection [34].

### From Early to Late SCV

Vacuolar bacteria are only able to multiply after a maturation phase of the vacuole marked by the sequential acquisition of different protein and lipid membrane markers. Within minutes of formation, Early SCVs display markers such as the EEA1 protein, the transferrin receptor [56] or the small GTPases Rab5 [57] and Rab4 [58] on their surface (**Fig. 1**), indicating interaction with early endosomal compartments. Maturation of SCVs is characterized by the loss of these early markers and the progressive acquisition of late endosomal markers such as the lysosomal glycoproteins LAMP1 and LAMP2 and the vacuolar ATPase (**Fig. 1**) [56]. At this stage, the so-called Late SCVs have a membrane composition similar to that of lysosomes. However, this is not the result of a direct fusion of SCVs with pre-existing lysosomes, since lysosomal hydrolases are excluded [59]. The GTPase Rab7, present on SCVs during the maturation phase, is also found on vesicles rich in lysosomal glycoproteins and poor in lysosomal enzymes that accumulate in the vicinity of SCVs and participate in their maturation [60].

The maturation of SCVs is thus distinct from that of phagolysosomes by the absence of fusion with pre-existing lysosomes. This segregation from the endocytosis pathway is an active process that depends on the synthesis of bacterial proteins [61, 62]. T3SS-1 and -2 and their associated effectors are intimately linked to *Salmonella* cell fate. However, their involvement in the maturation of SCV is not clear. A study by Garvis *et al*. in murine macrophages [62] showed that the ability of SCV to avoid phagolysosome-like maturation is not dependent on either the expression of a functional T3SS-2 or the *spv* operon of the virulence plasmid. In a study in epithelial cells, we showed that a *Salmonella* mutant with no functional T3SS-1 exhibits a very different pattern of acquisition of the EEA1 and LAMP1 markers within two hours of internalization compared to the wild-type strain [63]. This suggests that T3SS-1 plays a role in the maturation of SCVs. Two studies have highlighted the particular role of SipA in this phenomenon. This T3SS-1 effector whose expression persists even after the onset of T3SS-2 effector expression, sustains bacterial replication in both fibroblasts and macrophages [64]. A more recent study using a neonatal mouse infection model confirmed the importance of SipA. Zhang *et al*. showed that certain effectors of T3SS-1 are required for intracellular proliferation in small intestinal epithelial cells and that the absence of SipA causes a defect in vacuole maturation marked by the absence of the LAMP1 marker [65].

## FORMATION OF THE *SALMONELLA* REPLICATIVE COMPARTEMENT

The membrane protein composition of Late SCVs stabilizes, as far as can be judged, between 60 and 90 min after their formation. However, beyond this time, a strong increase in the presence of LBPA [66], a lipid specific to late endosomes [67], and cholesterol [66, 68] associated with SCVs is observed, showing that maturation is not complete. In addition, bacterial replication does not begin until 3–4 h after entry and coincides with the appearance of membrane tubules (**Fig. 1**; SITs, see following paragraphs) that emanate from the vacuole and extend throughout the cell. The presence of tubules is indicative of the implementation of T3SS-2 and the translocation of associated effectors that are expressed in response to the environment of the Late vacuole [69]. As previously mentioned, T3SS-2 and its effectors are essential for intracellular replication. The effectors, through their multiple activities, profoundly modify the biology of the infected cells (for review [70]) and have an important impact on the establishment of the replicative compartment of *Salmonella*.

### SifA blocks the transport of lysosomal enzymes

Like Late SCVs, the *Salmonella* replicative compartment is rich in lysosomal membrane glycoproteins and poor in lysosomal enzymes. This is due to the ability of *Salmonella* to block intracellular transport of lysosomal hydrolases via a T3SS-2 activity. The newly synthesized lysosomal enzymes bind and are transported by M6PRs (there are two different ones) from the *trans*-Golgi to the late endosomes/lysosomes. The receptors, freed from their ligand, then return to the *trans*-Golgi network (TGN) [71] by a transport process that requires the GTPase Rab9 [72]. In *Salmonella* infected cells, the T3SS-2 effector SifA, present on the surface of SCVs, sequesters Rab9. In the absence of available Rab9, the M6PRs remain trapped in recycling vesicles and are no longer available for the transport of newly synthesized lysosomal enzymes [73]. This leads to a misdelivery of the enzymes and to a depletion of the hydrolase content of lysosomes. SCVs can then exchange their contents with endocytic compartments, as appears to be the case [74, 75], while maintaining a level of lysosomal enzymes compatible with bacterial replication.

### *Salmonella*-induced tubules

#### There are different types of Salmonella-induced tubules

The presence of membrane tubules stretching from the SCV towards the cell periphery is a hallmark of *Salmonella*-infected cells (**Fig. 1**, **2A** and **4**). These structures were first identified by Gracia-del Portillo *et al*. [76] and named *Salmonella*-induced filaments (SIF). Since the discovery, the existence of other tubules has been demonstrated (for review see [77]). They have in common that they are formed from the *Salmonella* replicative compartment, require the expression of T3SS-2 and have T3SS-2 effectors on their surface. SIFs are, like SCVs, characterized by their high content of lysosomal glycoproteins and lipids (LAMP1, LAMP2, vATPase, LBPA, cholesterol) that reflects, as it is now generally agreed, their formation by the fusion of nascent tubules with late endocytic compartments [75].

In a screen for host proteins involved in *Salmonella* infection, it was found that certain resident proteins of the TGN and sorting/recycling endosomes are present and involved in the formation of tubules [78]. These are secretory carrier membrane proteins (SCAMPs) 2 and 3. The presence of these proteins defines a class of tubules that lack lysosomal glycoproteins and have been called SISTs for *Salmonella*-induced SCAMP3 tubules. The presence of SCAMPs and the strong inhibition of SISTs formation by Brefeldin A show, on the one hand, that there are strong interactions between SCVs and the TGN and, on the other hand, that the process of formation of these tubules is different from that of SIFs since the latter are resistant to the action of Brefeldin A.

The T3SS-2 effector SifA has a particularly important role in the formation of tubules since SIFs or SISTs are not observed in its absence [78, 79]. However, tubules that have the distinctive feature of being largely devoid of host proteins can be observed in cells infected with a strain that expresses neither SifA nor SopD2, another effector of T3SS-2. They are called LNTs for LAMP1-negative tubules and can only be visualized by the presence on their surface of other T3SS-2 effectors such as SseJ or PipB2 [75].

These different structures are now more generally referred to as *Salmonella*-induced tubules (SIT), a term that considers the globality of tubules of different composition that have been identified [77].

#### Formation and structure of Salmonella-induced tubules

Membrane tubules for exchange and transport between compartments are commonly observed in uninfected cells [80]. They serve, for example, in antigen-presenting cells for the transport of peptide-loaded class II molecules to the cell surface [81] or, in the endosomal compartment, for the sorting of molecules to be recycled to the *trans*-Golgi (for review see [82]). SITs are distinct from these transport structures by their substantial length and stability over time. They are easily observed in epithelial cells and have long been thought to be the preserve of these cells. This is not the case, however, as these structures have been observed, after infection, in interferon-gamma activated RAW264.7 mouse macrophages or bone marrow-derived dendritic cells [83].

Two studies published in 2008, using live cell imaging approaches, showed that LAMP1-positive SITs are highly dynamic structures. The nascent SITs stretch from the surface of the SCVs and have a bidirectional movement that is dependent on microtubules. The speed of extension/retraction of the tubules is maximal at the onset of their formation (about 4 hours after the start of infection) and decreases as the number and length of SITs increase. Later (beyond 8 hours) they branch, fuse with other tubules and eventually form a complex network throughout the infected cell [83, 84]. The formation of these structures depends on the cytoskeleton of the infected cell and in particular on the microtubules and their associated motors. SITs are supported by and extend along the microtubules that are essential for their formation [76]. However, once formed, they maintain their structure even in the absence of microtubules, but lose all dynamics [84].

A study using advanced imaging techniques has made significant progress in understanding the structure of SITs and discovered unsuspected complexity [85]. It revealed the presence of two structurally distinct types of SITs, both in epithelial cells and in macrophages. The first (diameter: 120±46 nm) are delimited by a single membrane and the content of their lumen is reminiscent to that of endocytic compartments. The second (diameters: 221±65 nm), are composed of two membranes. The luminal space between the inner and outer membranes is continuous with the SCVs and the inner lumen contains elements of the host cell cytosol and the cytoskeleton, in particular actin filaments and microtubules. This work proposes a model in which 1) single membrane SITs are the precursor tubules that develop from SCVs and 2) two membranes SITS are formed by a process of longitudinal invagination of the membrane of single membrane SITs. This process depends on the T3SS-2 effectors SseF and SseG.

#### Role of Salmonella-induced tubules in infection

The role of SITs has been questioned for a long time, certainly because these structures have not yet been observed *in vivo*. However, there are numerous correlative results and, recently, more direct evidence of the involvement of these structures in the intracellular replication of *Salmonella*. It is common to observe, at least *ex vivo*, several dozen *Salmonella* per cell in the late stages of infection. This replication confronts bacteria with two challenges which are obtaining the nutrients necessary for this growth and also sufficient membrane to keep each bacterium in an individual vacuole [86]. The role of SITs is to facilitate the access of *Salmonella* to the nutrients and to the membrane.

A number of T3SS-2 effectors control the formation and structure of SITs: SifA and PipB2 are necessary for their formation [79] and elongation [87], respectively; in the absence of SopD2, SITs are fewer, shorter, and show a discontinuous distribution of LAMP1 [88]; SITs of altered structures (called pseudo-SIFs) are observed in the absence of SseF or SseG [89]. Strains lacking either of these effectors exhibit attenuated virulence in murine models of infection and/or a replication defect in cultured cells. There is thus a correlation between the presence of intact SITs and the intracellular replication capacity of *Salmonella*. Another example of this correlation is provided by LNTs. A Δ*sifA* mutant, which is unable to induce the formation of SITs, shows a very marked replication defect in macrophages and a very strong attenuation of virulence in the mouse infection model. A strain that is additionally deleted from *sopD2* (Δ*sifA* Δ*sopD2*), which does produce LNTs, has a much higher replication capacity in macrophages and is more virulent than the Δ*sifA* mutant [75]. LNTs, like the tubules produced by the wild-type *Salmonella* strain, have the ability to wrap around host endocytic compartments, which most certainly promotes exchange and gives *Salmonella* access to endocytosed material. This ability to acquire content by fusion of SITs with compartments of the endocytic pathway has been documented by live cell imaging [84].

Importantly, it was recently demonstrated that membranes and lumen of SCVs and SITs form a continuum and their contents are rapidly exchanged [90]. The metabolic activity of bacteria is higher when their SCVs are connected to SITs, both in HeLa epithelial cells and in interferon-gamma-treated macrophages. Therefore, the fusion properties of SITs and their ability to rapidly exchange with SCVs allow *Samonella* to access the contents of endocytic and exocytic pathway compartments and promote their replication.

### Stability of the *Salmonella* replicative compartment

As seen earlier, the nascent vacuole is fragile and prone to rupture. But there are other circumstances that can destabilize vacuoles. This is the case for SCVs enclosing *Salmonella* not expressing SifA. After 4 hours of infection, Δ*sifA* SCVs progressively lose their lysosomal glycoproteins, are weakened and a continuous increase in the number of these mutant bacteria in the cytosol is observed [91]. It is likely that the inability of *Salmonella* to replicate in the cytosol of infected cells (with the exception of epithelial cells) is responsible for the high attenuation of a Δ*sifA* mutant in a mouse model of infection [79]. The decrease in lysosomal markers and the concomitant appearance of marked fragility of Δ*sifA* SCVs reflect the crucial role played by SifA in membrane exchanges between the *Salmonella* replicative compartment and late endosomal compartments of the host [92]. Indeed, this effector is essential for the recruitment of host proteins required for membrane tethering and fusion (see following chapters) and its absence compromises the ability of SCVs to perform the membrane exchanges required for its maintenance. It was also shown that vacuole rupture involves at least two other effectors of T3SS-2, SopD2 and SseJ. We have previously discussed the presence of LNTs associated with *Salmonella* Δ*sifA* Δ*sopD2* SCVs. These tubules exchange with host compartments, resulting in higher levels of lysosomal glycoproteins and better stability of SCVs [75]. A Δ*sifA* Δ*sseJ* mutant also resides in a more stable vacuole than the Δ*sifA* mutant [93]. SseJ is an enzyme with a lipid diacylation activity that modifies and stabilizes the SCV membrane [94]. However, unlike the previous mutant (Δ*sifA* Δ*sopD2*), a Δ*sifA* Δ*sseJ* mutant is more attenuated than a Δ*sifA* mutant, suggesting that the properties of its vacuole are abnormal, and do not support bacterial growth. This may be related to the role of SseJ in activating a signalling pathway that increases cellular cholesterol and improves intracellular survival of *Salmonella* [95].

### The fate of SCVs during bacterial replication and host cell mitosis

A particularity of SCVs is that they divide at the same time as the bacteria, maintaining *Salmonella* in an individual vacuole while many other intracellular bacteria, such as Chlamydia or Coxiella, mutualize their vacuole (for a review of this topic see [96]). It is very possible that the mechanisms of SCV division and SIT formation are partly similar since the formation of vacuoles containing multiple bacteria is frequently associated with a defect in SIT formation. The advantage to *Salmonella* of residing alone in a vacuole is somewhat enigmatic but it has been proposed that this allows each bacterium to have less competition for access to nutrition while increasing the number of targets, allowing a dilution of immune defense mechanisms [86].

When the infected cell divides, the SCVs are in most cases present in both daughter cells but show an asymmetric distribution, i.e. one daughter cell receives more SCVs than the other. During cytokinesis, the SCVs of either daughter cell remain connected by SITs until the very last moment (**Fig. 2C**) and effectors of T3SS-2 that are involved in SIT formation (SifA, SopD2 and SspH2) promote the presence of bacteria in both daughter cells [97]. The distribution of organelles or chromosomes during mitosis is usually symmetrical. However, asymmetric distribution may play a physiological role. For example, mitochondria are distributed asymmetrically and according to their functional state during mitosis of human mammary stem cells. This prevents the transfer of damaged mitochondria to the daughter cell intended to become a new stem cell [98]. What about the asymmetric distribution of SCVs? Overall, these processes reduce the bacterial load of one of the daughters and thus may contribute to its survival. However, it remains difficult to assess the impact of these observations on the infection of an organism.

## IMPORTANCE OF THE HOST CELL CYTOSKELETON FOR THE FORMATION OF THE *SALMONELLA* REPLICATIVE COMPARTEMENT

The cytoskeleton is responsible for the mechanical properties of the host cell, giving it its shape and allowing it to deform and move. It is also essential for intracellular transport, particularly of membrane compartments and their exchanges. In eukaryotic cells, it consists of actin filaments, microtubules and intermediate filaments. Actin filaments are actin polymers, mainly located under the plasma membrane. They allow the deformations of the plasma membrane responsible for the movement of cells, the adhesion, or the deformation of the membrane necessary for phagocytosis (e.g. of *Salmonella*). Microtubules are polymers of α-tubulin and β-tubulin, which can be several tens of μm long. They are polarized and, in most cells, organized from the microtubule organizing center (MTOC), located near the nucleus, and radiate into the cell in an umbrella-like organization. Intermediate filaments are the most stable structures and their protein composition varies according to cell type. Molecular motors use the cytoskeleton as tracks to transport organelles, vesicles, nuclear material or protein complexes. Myosins move along actin filaments. Dynein and kinesins move along the microtubules, with dynein moving towards the (-) ends and most kinesins towards the (+) ends.

Actin filaments are manipulated by T3SS-1 effectors for invasion of non-phagocytic cells (see previous chapter). This chapter focuses on the use and manipulation of the host cell cytoskeleton for the movement, localization and membrane dynamics of SCVs and SITs.

### The nascent SCV recruits dynein 

**Figure 3:**
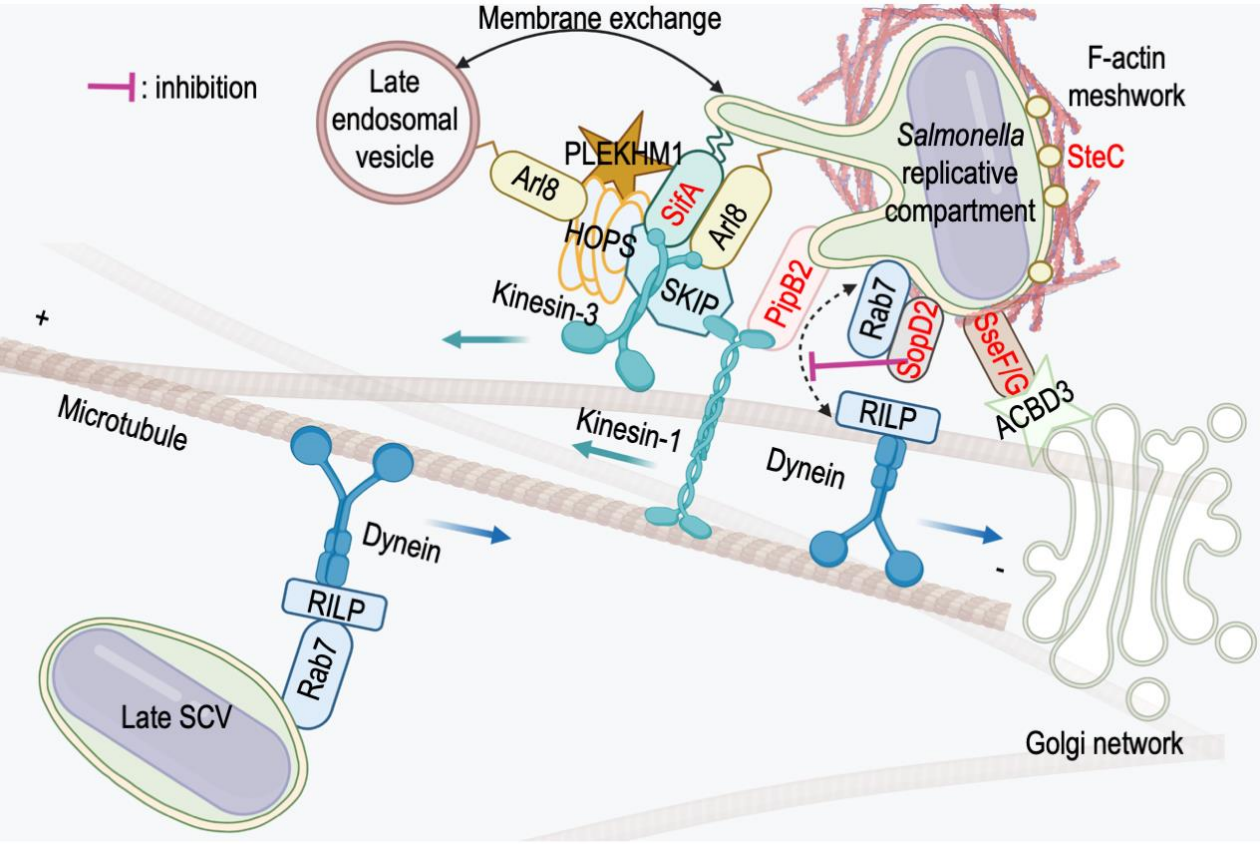
FIGURE 3: *Salmonella* effectors and host proteins interactions. The small GTPase Rab7 is acquired during the maturation phase of the SCV and its presence is persistent thereafter. Its association with RILP allows the recruitment of dynein and the retrograde movement of the maturing Late SCV to the juxtanuclear region. Specific T3SS-2 effectors (in red) are localized on the replicative compartment. The presence of SopD2 maintains Rab7 in a GDP-bound form that does not bind RILP and prevents dynein recruitment. SteC has a kinase activity necessary for actin polymerization in the vicinity of SCVs/SITs. SseF/G anchor the SCV to the Golgi network via an interaction with the Golgi resident protein ACBD3. Both PipB2 and SifA recruit kinesin-1, respectively through a direct interaction or via SKIP. Kinesin-3 is recruited through its interaction with SifA and SKIP. SifA recruits the small GTPase Arl8 and these proteins are bound to the membrane by geranylgeranylation or acetylation, respectively. SKIP and PLEKHM1 compete to bind to SifA and Arl8 and both recruit the HOPS tethering complex on SCVs/SITs to initiate the fusion process with the late membrane compartments of the host cell.

Maturation of the SCV is characterized by changes in the composition of the vacuole membrane but also by a change in intracellular localization. During the first hours of infection, the SCV moves from the site of entry to the MTOC in the juxtanuclear region (**Fig. 1** and **3**). This retrograde movement is mediated by cytoplasmic dynein [99, 100], a very large polypeptide complex, with a molecular weight above 1 MDa, and composed of dozens of subunits. Dynein uses ATPase activity to move on microtubules (for review see [101]). The engagement of this molecular motor is controlled by the small GTPase Rab7, which is recruited during the maturation of the SCV. The interaction is indirect, mediated by its effector Rab7-interacting lysosomal protein (RILP) [102–104].

In the later stages of infection, the presence of dynein is more difficult to detect. The *Salmonella* replicative compartment and SITs appear to lack it, despite the presence of Rab7 [66]. However, the involvement of the molecular motor is undeniable because SITs are endowed with a bidirectional movement on microtubules (see previous chapter), because the formation of SITs is strongly affected when the activity of dynein is inhibited [99] and finally because the presence of dynein on SCVs, implicating the T3SS-2 effector SseF, has been reported [105]. However, it seems that the Rab7-RILP-dynein association is in fact transient and regulated. In a 2004 paper, Harrison *et al*. [100] show that the effector SifA interacts with Rab7 and proposed that this interaction is responsible for the uncoupling of Rab7 from RILP. This phenomenon could also involve other effectors. D’Costa *et al*. [106] have shown that SopD2 binds to Rab7 but the binding site is different from that of RILP, which rules out uncoupling by binding competition. The interaction with SopD2 blocks Rab7 nucleotide exchange and limits the recruitment of RILP (**Fig. 3**), which interacts preferentially with the GTP-bound form of this GTPase.

At later stages of infection, dynein recruitment might also be regulated in a different way. Based on the BioID screen, a recent study [107] reported that HPS3 and HPS5, which are two of the three subunits of BLOC-2 (Biogenesis of Lysosome-related Organelles Complex 2), interact with SifA. The third subunit of this complex, HPS6, interacts directly with a subunit of the dynein motor complex and links this retrograde molecular motor to the lysosome [108]. In infected cells, BLOC-2 regulates both the positioning and the stability of SCVs and this suggests that dynein is involved.

### The *Salmonella* replicative compartment recruits kinesins

There is a wide variety of kinesins and these molecular motors are involved in a large number of cellular processes [109]. The kinesin superfamily consists of 14 families named kinesin-1 to kinesin-14 [110]. These motors share a common motor domain, usually N-terminal, and show strong variations in cargo binding domain and their quaternary structure. A siRNA screen of about 30 molecules showed the involvement of several kinesins (KIF5B, KIFC1, KIF11 and KIF24) in the formation of SITs [111]. However, KIF5B (kinesin-1 heavy chain) and KIF1A and KIF1Bß, which belong to the kinesin-3 family, are the only motors whose involvement has really been specified.

#### PipB2 is a linker for kinesin-1

**Figure 4:**
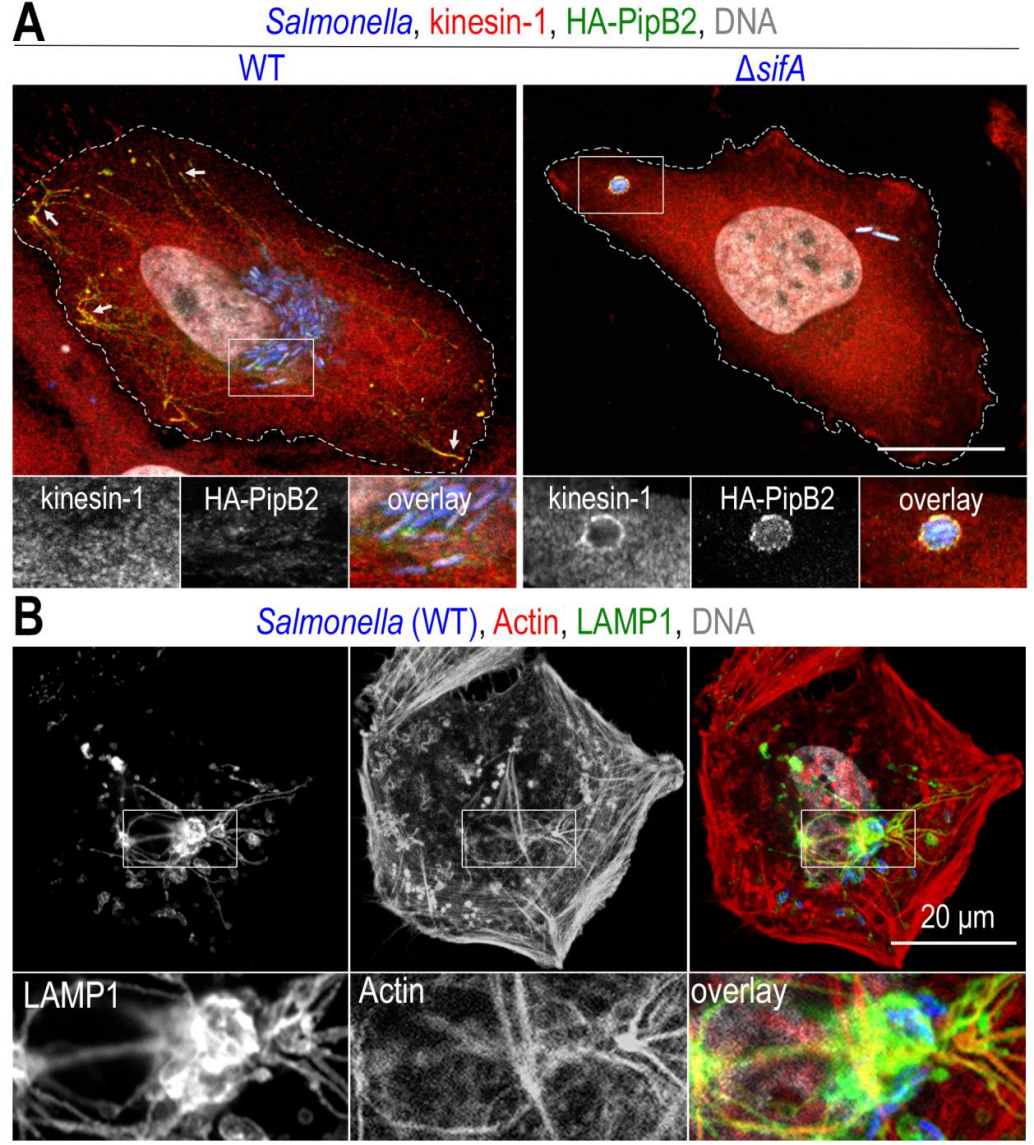
FIGURE 4: Recruitment of molecular motors and the actin cytoskeleton on *Salmonella* vacuoles. Confocal microscopy images, after immunolabelling, of *Salmonella*-infected HeLa (**A**) or Cos-7 (**B**) cells at a late time (12h) of infection. (**A**) Two effectors of T3SS-2, SifA and PipB2 recruit kinesin-1 to SCVs and SITs. The presence of kinesin-1 (red) and PipB2 (green) is detected at the tip of SITs (arrows) in cells infected with wild-type (WT) *Salmonella* (blue), while these molecules are difficult to detect on SCVs (see inset). In the absence of SifA (Δ*sifA*), kinesin-1 and membrane effectors accumulate on SCVs. (**B**) Actin labelling (red) highlights the presence of a strong actin cortex. Actin is also present around the juxtanuclear *Salmonella* microcolony (blue) and also marks the SIT network (LAMP1, in green). Scale bars, 20 or 10 μm for the magnified insets in (**A**) and 6.7 μm for the magnified insets in (**B**). This figure is composed of unpublished images.

Kinesin-1 is a heterotetramer consisting of two heavy chains (KHC, encoded by KIF5A, KIF5B or KIF5C) and two light chains (KLC, encoded by KLC1-4). The motor domain is located in the N-terminal part of the KHCs and the KLCs bind to the cargo [112]. As in the case of dynein, the presence of kinesin-1 on bacterial compartments is difficult to detect by light microscopy and was first revealed indirectly and then through the analysis of a *Salmonella* mutant. In 2004, two studies showed that treatment of infected cells with aurintricarboxylic acid, a potent kinesin inhibitor, inhibits *Salmonella* replication [99] and SIT formation [100]. Similarly, overexpression in infected cells of the cargo-binding domain of KLC2, which has a dominant negative effect on kinesin-1 activity, decreases the number of SITs [100]. Finally, the study of a Δ*sifA* mutant that we conducted in 2005 showed that the localization of this mutant, more peripheral than wild-type bacteria, is due to the accumulation of kinesin-1 on the SCV (**Fig. 4A**) [113]. Based on this observation, by deleting genes encoding other T3SS-2 effectors in a Δ*sifA* mutant, we showed that PipB2 [114] is responsible for the recruitment of kinesin-1 through a direct interaction with KLCs (**Fig. 3**) [115].

Previously, Knodler *et al*. showed the appearance of abnormally short SITs in cells infected with a Δ*pipB2* mutant [87]. The re-reading of this article in the light of the discovery of the interaction of PipB2 with KLC2 suggests that PipB2-mediated recruitment of kinesin-1 allows the elongation of SITs along microtubules. This was confirmed by a live cell study showing that a Δ*pipB2* mutant induces the formation of large, non-dynamic SITs [116]. In a recent study using immortalized macrophages from C57BL/6 wild-type or KIF5B^−/−^ mice, we showed that kinesin-1 also participates in the positioning of SCVs, keeping them in the juxtanuclear region but distant from the nucleus [117]. The molecular determinants of PipB2 that allow interaction with KLC are still unknown. This protein of 350 amino acid residues has a C-terminal pentapeptide sequence (L^341^FNEF^345^) that is required for the redistribution of lysosomes to the cell periphery upon ectopic expression or, in infected cells, the accumulation of LAMP1-positive vesicles at the cellular periphery [87]. However, this sequence is not required for interaction with kinesin-1 [115].

Cellular kinesin-1 is for the most part in an auto-inhibited conformation. This state involves interactions between KHC and KLC and allows this motor to be used only when necessary. In an *in vitro* assay using microtubule gliding or reconstitution of membrane tubules from artificial vesicles, we have shown that interaction with PipB2 is sufficient to relieve the inhibition of kinesin-1, which then engages and moves on microtubules [117]. However, the situation is more complicated in infected cells where this process involves several effectors and host proteins.

#### SifA recruits kinesin-1 through SKIP

As mentioned earlier, the function of PipB2 was identified through the accumulation of kinesin-1 observed in the absence of SifA. This effector is itself involved in the recruitment and regulation of kinesin-1 activity as it interacts with the host protein SKIP (SifA and kinesin-interacting protein) which binds kinesin-1 (**Fig. 3**) [113]. SifA is essential for the formation of SITs [79] and, as seen above, its absence confers other unusual membrane properties to SCVs. However, the accumulation of kinesin-1 on Δ*sifA* SCV (**Fig. 4A**) does not appear to be the cause of vacuole fragility because that of a Δ*sifA* Δ*pipB2* mutant, lacking kinesin-1, is equally fragile [77].

SKIP is an important mediator of SifA functions as some of the phenotypes observed in the absence of SifA are also observed in cells invalidated for SKIP expression (siRNA or Knockout). These include the absence of SITs and the accumulation of kinesin-1 on SCVs [92, 113]. These two phenotypes are probably related since the absence of SITs reduces the membrane surface of the bacterial compartment and concentrates the kinesin-1 recruited by PipB2 on the SCV. Under these conditions, kinesin-1 does not seem to be very active as evidenced by the position of the Δ*sifA* SCVs, which are often located at the midpoint between the nucleus and the plasma membrane. However, the low activity of the molecular motor is probably not (solely) responsible for the absence of SITs [118]. Based on these observations, we have proposed that SKIP might be essential for the activation of PipB2-recruited kinesin-1 [119]. A recent study supported this hypothesis by showing that interactions of the N-terminal part of SKIP with the light and heavy chains of kinesin-1 promote activation of the molecular motor [120].

#### SifA and SKIP recruits KIF1A and KIF1Bß

In a recent study [118] we showed that KIF1A and KIF1Bß, highly homologous proteins of the kinesin-3 family, are recruited to SCVs and SITs. These molecular motors are important for the transport of lysosomes and synaptic vesicle precursors and are recruited to membranes by the lysosomal GTPases Arl8 [121, 122]. In *Salmonella*-infected cells, their recruitment involves interactions with the SifA/SKIP complex (**Fig. 3**). Silencing of KIF1Bß indicated that this motor is involved in the positioning of SCVs and is required for the formation of SITs by acting upstream of kinesin-1. It is also important for the stability of SCVs.

#### SifA interacts with the small GTPase Arl8b

Resolution of the crystal structure of SifA complexed to the PH domain of SKIP has shown that this protein consists of two distinct domains [123, 124]. The N-terminal domain interacts with SKIP. The C-terminal domain has structural similarity to the *Salmonella* effector SopE which has Rho GTPase guanine nucleotide exchange factor (GEF) activity. Purified SifA, however, did not show guanine nucleotide exchange activity for the RhoA GTPase to which it does bind [123, 124].

It is not yet known whether SifA actually has GEF activity and if so, towards which GTPase. In a recent work [125] we showed that the small lysosomal GTPases Arl8a/b [126, 127] are possible candidates. Both domains of SifA interact with Arl8b and the effector promotes its recruitment to SCVs. Outside the infectious context, Arl8a/b interacts with SKIP and this complex is essential for the recruitment of kinesin-1 and KIF1A/KIF1Bß to lysosomes [122, 128]. Thus, on *Salmonella* compartments, SifA and Arl8b form a complex and recruit SKIP and kinesins in parallel [118].

### The actin cytoskeleton encloses the replicative compartment of *Salmonella*

As seen previously, T3SS-1 effectors manipulate the actin cytoskeleton to facilitate invasion of non-phagocytic cells. The T3SS-2 effectors also do this with a very different result. In a study published in 2001, we showed that, during the replication phase, SCVs are very frequently surrounded by actin filaments [129]. This can take the shape of short actin filaments forming cylindrical structures rising perpendicularly from the basement membrane and enclosing the SCVs [129] or condensed clusters of actin around which elements of the microcolony are distributed [130]. Although not essential for their formation [131], actin is also found on SITs (**Fig. 4B**).

Several effectors of T3SS-2 participate in the presence of actin around the *Salmonella* replicative compartment. SteC is essential for this phenotype [132, 133]. This effector has sequence similarities to the human kinase Raf-1 and its kinase activity is required for actin polymerization. Several substrates of SteC, involved at different levels of actin filament formation, have been identified (for a full review of this topic, see [134]). SspH2 and SseI both interact with filamin and colocalize with cortical actin upon ectopic expression [130]. SspH2 also interacts with profilin and colocalizes with actin surrounding SCVs. Finally, the same study shows that SpvB, which has since been shown to be a substrate for T3SS-2 [135], inhibits the polymerization of SCV-associated actin through its enzymatic activity. The presence of effectors that can activate or inhibit SCV-associated actin suggests a regulation whose modalities remain to be defined. The role of actin associated with the *Salmonella* replicative compartment remains poorly understood. Intracellular replication studies of a mutant not expressing SteC and virulence studies in animals have given variable results but tend to show that this effector limits the intracellular growth of *Salmonella* [132]. Heggie *et al*. proposed that SteC plays a role in the intestinal phase of salmonellosis since this effector is well conserved in *Salmonella* strains that cause gastrointestinal disease and homologues of this effector are present in other bacteria with intestinal tropism [133].

## THE MEMBRANE FUSION MACHINERY AND THE *SALMONELLA* REPLICATIVE COMPARTEMENT

### The HOPS tethering complex

Fusion processes allow the exchange of contents between membrane compartments. In *Salmonella*-infected cells, they take place throughout the intracellular course of the bacterium, from maturation of the nascent SCV to vesicular recruitment mediated by SITs that ensure the flow of nutrients essential for intracellular bacterial replication. In eukaryotic cells, they are supported by tethering complexes whose function is to bring together SNARE proteins present on opposing membranes to initiate the fusion process. The tethering complexes of the endocytic pathway, CORVET and HOPS, are hetero-hexamers that share four subunits and are recruited to the membranes through their interactions with small GTPases. The HOPS complex (for review see [136]) consists of a class C subset of vacuolar protein sorting (Vps) proteins (Vps11, Vps16, Vps18 and Vps33) and two HOPS-specific proteins, Vps39 and Vps41, which are required for fusion of vesicles from the late endocytic pathway [137, 138].

### *Salmonella* recruits the HOPS tethering complex to its replicative compartment

In recent years, several studies have revealed multiple interactions that allow the recruitment of the HOPS complex to the *Salmonella* replicative compartment. SKIP, recruited by SifA, interacts with the Vps39 subunit [139] allowing the assembly of other subunits of the HOPS complex on SCVs and SITs [140]. This assembly is also dependent on Arl8b whose recruitment is itself mediated by SifA [125]. It involves an interaction of Vps41 and Arl8b, which outside of the infectious context, participates in the assembly of the HOPS complex on lysosomes [139]. PLEKHM1 is a homologous protein of SKIP (SKIP is also called PLEKHM2). These proteins share a similar domain organization and numerous interactors. Like SKIP, PLEKHM1 is recruited to the *Salmonella* replicative compartment via a direct interaction with SifA [141]. PLEKHM1 was originally described as an effector of Rab7 [141, 142]. However, it also interacts with Arl8 [143] and competes with SKIP for the binding of Arl8b and SifA [143]. PLEKHM1 binds the HOPS subunits Vps39 and Vps41 via its RUN domain [144]. Finally, a study of effector-host interactions during infection has recently identified direct, probably transient, interactions between SifA and Vps39 [145].

Recruitment of the tethering complex to the *Salmonella* replicative compartment is thus orchestrated by SifA, through its various interactions with host proteins. This explains the strong phenotypes associated with the absence of SifA. The progressive loss of lysosomal membrane proteins associated with Δ*sifA* SCVs certainly reflects the loss or, at least, a very strong decrease in membrane fusion capabilities whereas the phenotypes associated with the absence of SKIP [113], PLEKHM1 [141] or Arl8 [111] are less pronounced and testify to a partial loss of the capacity to recruit the HOPS complex.

## CONCLUSION

Although sometimes present in the cytosol of epithelial cells, *Salmonella* adopts an intravacuolar mode of life during their infectious cycle. This review illustrates the importance of T3SS effectors in the tailoring and maintenance of the membrane constituting the intracellular replicative niche and more particularly that of SifA. This T3SS-2 effector is a pillar that allows the anchoring of host proteins which, in turn, possess multiple interactors capable of regulating the membrane dynamics of SCVs and SITs. Molecular motors are, in this respect, important players. Kinesin-1 is necessary for the elongation of SITs. It is also sufficient, recruited by PipB2, for the extrusion of tubules from artificial lipid vesicles. However, in infected cells, this motor is not essential for their formation. In this context, we have recently found that kinesin-3 is also required for tubule formation by acting upstream of kinesin-1 (Fang *et al*., in press) [118]. Kaniuk *et al*. [111] showed that silencing of motors other than kinesin-1 or kinesin-3 affected the capacity of tubule formation in infected cells. Future studies will undoubtedly identify them and reveal their mechanisms of regulation and action. Although difficult to detect, dynein is also an actor whose activity is regulated during infection by T3SS-2 effectors. However, the recruitment and role of this retrograde motor is still unclear. Two preprints [146, 147] show that the proteins RUFY3 and RUFY4 interact with Arl8 and dynein and promote retrograde transport of lysosomes making them credible candidates for dynein recruitment to *Salmonella* compartments. It is also evident that other effectors, which obviously participate in the membrane dynamics of SCVs, influence the activity of molecular motors without their mode of action being understood. This is for example the case for SteA [148, 149] for which the phenotype resulting from the deletion is dependent on kinesin-1 or dynein activity.

## Abbreviations:

GEF: – guanine nucleotide exchange factor;
KHC: – kinesin heavy chain;
KLC: – kinesin light chain;
LNT: – LAMP1 negative tubule,
M6PR: – mannose-6 phosphate receptor;
M cells: – microfold cells;
MTOC: – microtubule organizing center;
RILP: – Rab7-interacting lysosomal protein;
SCAMP: – secretory carrier membrane protein;
SCV: – Salmonella-containing vacuole;
SIF: – Salmonella-induced filament;
SIST: – Salmonella-induced SCAMP3 tubule;
SVAT: – spacious vacuole-associated tubules;
T3SS: – type 3 secretion system;
TGN: – trans-Golgi network.
